# Parental liver disease mortality is associated with unfavorable outcomes in patients with alcohol-associated hepatitis

**DOI:** 10.1097/HC9.0000000000000666

**Published:** 2025-05-23

**Authors:** Wanzhu Tu, Samer Gawrieh, Lauren Nephew, Craig McClain, Qing Tang, Srinivasan Dasarathy, Vatsalya Vatsalya, Douglas A. Simonetto, Carla Kettler, Gyongyi Szabo, Bruce Barton, Yunpeng Yu, Patrick S. Kamath, Arun J. Sanyal, Laura Nagy, Mack C. Mitchell, Suthat Liangpunsakul, Vijay H. Shah, Naga Chalasani, Ramon Bataller

**Affiliations:** 1 Department of Medicine, and Department of Biostatistics and Health Data Science, Indiana University School of Medicine, Indianapolis, Indiana, USA; 2 Department of Medicine, University of Louisville, Louisville, Kentucky, USA; 3 Department of Gastroenterology, Hepatology and Nutrition, Cleveland Clinic, Cleveland, Ohio, USA; 4 Department of Internal Medicine, Mayo Clinic, Rochester, Minnesota, USA; 5 Department of Medicine, Harvard University, Boston, Massachusetts, USA; 6 Department of Population and Quantitative Health Sciences, University of Massachusetts, Worcester, Massachusetts, USA; 7 Department of Internal Medicine, Virginia Commonwealth University, Richmond, Virginia, USA; 8 Department of Internal Medicine, UT Southwestern Medical Center, Dallas, Texas, USA; 9 Department of Medicine, Universitat de Barcelona, Barcelona, Spain

**Keywords:** alcohol-associated hepatitis, alcohol use disorder, mortality

## Abstract

**Background:**

How parental alcohol use disorder and liver disease-related mortality influence the risk and the outcomes of alcohol-associated hepatitis (AH) in the offspring is unknown.

**Methods:**

We analyzed data from 2 prospective observational studies of AH cases and heavy drinking controls (HDCs). Family history of parental alcohol use disorder and liver disease mortality was assessed at the study entry. Logistic regression and Cox proportional hazard models were used to assess the influences of family history on AH development and outcome.

**Results:**

Data from 1356 participants in two prospective cohorts (926 AH cases and 430 HDC) were combined and analyzed. Parental alcohol use disorder was found in 56.9% of AH cases and 61.1% of HDC; parental death due to liver disease was reported in 7.5% of AH cases and 5.7% of HDC. Multivariable logistic regression showed that parental liver disease-related mortality was associated with more than a doubled risk of AH development in the offspring after controlling for their demographic characteristics and drinking behavior (OR=2.26, 95% CI: [1.22, 4.20]). Moreover, among the AH cases, having a parent die of liver disease significantly increased the 90-day mortality of study participants after adjusting for the effects of other risk factors (HR=2.26, 95% CI: [1.05, 4.86]).

**Conclusions:**

The study highlights the influences of parental death due to liver disease on AH development and mortality. Identifying patients at risk of AH through family history might help facilitate discussions on reducing alcohol consumption.

## STUDY HIGHLIGHTS

**What is known**

- Alcohol overconsumption is a fundamental contributor to the development of alcohol-associated hepatitis (AH). Individuals with a family history of alcohol use disorder (AUD) are at higher risk of becoming hazardous drinkers.
- Previous genetic studies have observed the associations between hereditary risk factors and liver cirrhosis. However, the effects of family history on AH development and mortality have not been studied.
- Multiple genes have been implicated in the risk of AUD. However, only a minority of individuals with AUD will develop AH.
- It is unclear how parental drinking and liver disease affect the AH risk in offspring.

**What is new here**

- The current study showed that compared to controls, AH cases were more than twice as likely to have a parent who died of liver disease.
- Identifying patients at high risk of AH, the most severe phenotype of alcohol-associated liver disease, might facilitate discussions on reducing alcohol consumption.

## INTRODUCTION

Alcohol-associated hepatitis (AH) is one of the deadliest forms of alcohol-associated liver disease (ALD). While overconsumption of alcohol is a fundamental contributor to AH, the precise mechanisms that turn an individual who drinks heavily into one with AH are not fully understood. Moreover, factors associated with AH are largely unknown. Genetic factors are known to influence the development of alcohol use disorder (AUD).^1^,^2^,^3^ Multiple studies of twins and siblings showed that genetic susceptibility conveyed a strong influence on alcohol sensitivity, addiction, and a broad range of drinking-related behaviors, either directly or indirectly, through their interactions with environmental factors.^4^,^5^,^6^,^7^,^8^ Hereditary disposition alone, however, does not seem to predetermine the risk of excessive alcohol use since individuals without a family history of alcohol misuse also develop AUD. Similarly, not all heavy drinkers sustain liver damage to the extent that gives rise to AH.^9^ The influences of genetic variants on individual susceptibility to advanced stages of ALD have been studied.^10^,^11^ Genome-wide association studies identified specific risk loci for alcohol-associated cirrhosis,^12^,^13^,^14^,^15^ although no study has specifically examined the hereditary effects on AH. Candidate gene studies showed variants in the patatin-like phospholipase domain-containing protein 3 (*PNPLA3*) and haptoglobin genes increased the risk and severity of AH.^16^ More recently, a prospective cohort study showed a significantly increased risk of advanced liver disease in first-degree relatives of those with metabolic dysfunction–associated steatotic liver disease.^17^ However, whether similar hereditary influences exist in AH has not been investigated.

Here, we hypothesized that the risk of AH was higher in individuals with a parent affected by AUD or one who died from advanced liver disease. We secondarily examined the survival of patients with AH with and without such a family history. To test these hypotheses, we analyzed data from 2 prospective cohorts comprised of patients with AH and heavy drinking control (HDC) subjects. The American Association for the Study of Liver Diseases (AASLD) and the U.S. Preventive Services Task Force (USPSTF) recommend exploring AUD in the diagnosis of ALD and implementing behavioral counseling intervention to address unhealthy alcohol use.^18^,^19^ By examining the associations between parental AUD/liver disease mortality and AH development and outcome in the offspring, this research stands to contribute to AH risk assessment and tailored management strategies.

## METHODS

### Study design and participants

This study was based on prospective observational data generated by 2 AH research consortia: The Translational Research Evolving Alcoholic Hepatitis Treatment (TREAT; participants recruited between June 2013 and December 2015) and the Alcoholic Hepatitis Network (AlcHepNet; participants recruited between July 2020 and December 2023). The TREAT consortium included 3 academic medical centers, as previously described.^20^ The AlcHepNet consortium consisted of 8 academic medical centers, and its observational cohort was recruited as a companion study to a randomized controlled clinical trial.^21^,^22^ TREAT was approved by the Institutional Review Board (IRB) at each participating institution, and AlcHepNet Observational Study was approved by a central IRB. All participants signed an informed consent before enrollment.

The TREAT and AlcHepNet are prospective observational studies targeting the same patient population. The 2 studies had a similar prospective design, used the same evaluation instruments, and were conducted under nearly identical protocols. In both studies, heavy drinkers with AH (cases) and heavy drinkers without significant liver disease (HDCs) were prospectively recruited. Heavy drinking was defined as regular consumption of alcohol with an intake of >40 mg daily or >280 mg weekly for women and >60 mg daily or 420 mg weekly for men for 6 months or more. The diagnosis of AH was made by the treating physician using the NIAAA consensus definition,^23^ as indicated by an onset of jaundice (serum total bilirubin >3 mg/dL within 8 weeks of presentation), history of heavy drinking with less than 8 weeks of abstinence before the onset of jaundice, AST >50 IU/L, AST/ALT >1.5 and both values <400 IU/L, and/or histological evidence of AH, and age ≥21 years. Medical history, physical exam, and liver enzyme levels were used to rule out liver disease in the HDCs. The studies excluded individuals with liver disease caused by hemochromatosis, autoimmune liver disease, Wilson disease, and acute viral hepatitis. Also excluded were pregnant or breastfeeding women and individuals with a history of liver transplants.

### Measures

The studies collected sociodemographic characteristics of the participants, including age, race/ethnicity, marital status, highest level of educational attainment, and current employment. Clinical characteristics assessed included vital signs, body mass index (BMI), liver disease history (measured through the Chronic Liver Disease questionnaires), and concomitant medications.

Alcohol use was evaluated by the Timeline Follow-Back (TLFB) and the Alcohol Use Disorder Identification Test (AUDIT-C) questionnaires.^24^,^25^ Both studies employed 2 TLBF queries over a 30-day retrospective period to assess participants’ recent alcohol use. The 2 questions were “Indicate the total number of drinks for the last 30 days” and “The total number of drinking days out of 30 days before the visit.”

Parental AUD was determined based on participants’ responses to the question, “Has your blood or natural father/mother been an alcoholic or problem drinker at ANY time in his or her life?” For those that answered “yes” to the parental AUD question, parental liver disease mortality was determined from participant responses to the question “Did he/she die of liver disease?” The studies did not ask about the specific types of parental liver diseases and causes of death, nor did they inquire about the disease and mortality status of other family members, such as siblings.

### Statistical analysis

All analyses were performed on combined data from the TREAT and AlcHepNet observational cohorts. We described the baseline sociodemographic and clinical characteristics of the study participants by AH case status. Categorical variables were compared using chi-square tests; numerical variables were compared using two-sample *t* tests. The proportions of participants who had a parent with a drinking problem or had a parent who died of liver disease in the case and control groups were compared using Chi-square tests. Logistic regression analyses were performed to assess the associations between the development of AH and parental drinking as well as parental liver disease mortality while controlling for the demographical and clinical characteristics of the study participants. Independent variables of primary interest in the logistic regression analysis were parental drinking and liver disease mortality. The final model included these 2 variables and other patient characteristics selected through a stepwise variable selection procedure. The model for AH risk controlled for participant’s age, sex, race, education, BMI, Hispanic ethnicity, total of drinks in the last 30 days, and parental AUD. A separate logistic regression analysis was used to assess the association between parental death due to liver disease and 90-day mortality among the AH cases. In this analysis, we controlled for age, sex, race, baseline MELD score, ALT, total white blood cell count, platelet count, and whether the patient received steroid treatment (yes or no). For both analyses, independent variable effects were expressed as OR and reported together with the associated 95% CIs. In a secondary analysis, we further conducted a Cox regression analysis to assess the associations between the family history variables and the overall survival of the patients with AH. Times to all-cause mortality in patients with AH who did not die were censored at the end of observation. Effects were expressed as HRs with corresponding 95% CIs. All analyses were performed in SAS software. *p* values <0.05 were considered statistically significant.

## RESULTS

### Patient characteristics

The derivation of the study cohort was presented in a flow diagram in Supplemental Figure S1, http://links.lww.com/HC9/B942. The baseline characteristics of the study participants, stratified by AH case status, are presented in (Table 1). Comparatively, AH cases were slightly younger (45.3±10.7 y in AH cases vs. 47.2±13.0 y in HDC; *p*<0.01) and had a higher proportion of White participants (84.0% vs. 76.3%; *p*<0.01), and more likely to be of Hispanic ethnicity (6.9% vs. 1.9%; *p*<0.01). Patients with AH were less likely to have education beyond high school (58.1% vs. 68.3%; *p*<0.01) and had a slightly higher BMI (29.6±7.5 vs. 28.4±6.9 kg/m^2^; *p*<0.01) compared to HDC, which appeared to represent a numerical rather than clinically meaningful difference. On average, patients with AH consumed significantly fewer drinks than the HDC in the 30 days before enrollment (185.5±21.3 vs. 301.1±277.8; *p*<0.01). Patient characteristics of the TREAT and AlcHepNet Cohorts were presented in Supplemental Table S1, http://links.lww.com/HC9/B942.

**TABLE 1:** Baseline characteristics of study participants

	Group			
Variable label	Overall N=1356	AH cases N=926	HDC N=430	*p*
Age at enrollment	45.9±11.5	45.3±10.7	47.2±13.0	0.006
Gender				0.782
Male	801 (59.6%)	543 (59.3%)	258 (60.1%)	
Race				<0.001
Non-White	250 (18.4%)	148 (16.0%)	102 (23.7%)	
White	1106 (81.6%)	778 (84.0%)	328 (76.3%)	
Ethnicity				<0.001
Hispanic or Latino	70 (5.3%)	62 (6.9%)	8 (1.9%)	
Non-Hispanic	1250 (94.7%)	835 (93.1%)	415 (98.1%)	
Highest level of formal education completed				<0.001
Elementary/year 10/high school	492 (38.5%)	358 (41.9%)	134 (31.7%)	
Trade school/college/graduate program	786 (61.5%)	497 (58.1%)	289 (68.3%)	
Are you currently employed?				0.181
No	542 (61.2%)	399 (62.5%)	143 (57.7%)	
Yes	344 (38.8%)	239 (37.5%)	105 (42.3%)	
BMI	29.2±7.3	29.6±7.5	28.4±6.9	0.006
Has your blood or natural father/mother been an alcoholic or problem drinker at ANY time in his/her				0.157
No	515 (41.8%)	359 (43.1%)	156 (38.9%)	
Yes	718 (58.2%)	473 (56.9%)	245 (61.1%)	
Did he/she die of liver disease?				0.259
No	1076 (93.1%)	728 (92.5%)	348 (94.3%)	
Yes	80 (6.9%)	59 (7.5%)	21 (5.7%)	
Indicate the total number of drinks for 30 days	225.1±243.6	185.5±213.3	301.1±277.8	<0.001
Indicate the total number of drinking days out of the 30 days prior to the visit	19.9±10.7	17.8±11.2	23.8±8.2	<0.001

Abbreviations: AH, alcohol-associated hepatitis; HDC, heavy drinking control.

The proportions of participants with parents who had AUD were similar to those with AH and HDC (56.9% in cases vs. 61.1% in controls; *p*=0.157). The proportion of participants with a parent who died of liver disease was higher in patients with AH than HDC (7.5% in cases vs. 5.7% in controls), but the difference in this direct comparison did not reach the level of statistical significance (*p*=0.26).

### Factors associated with AH development

**FIGURE 1:**
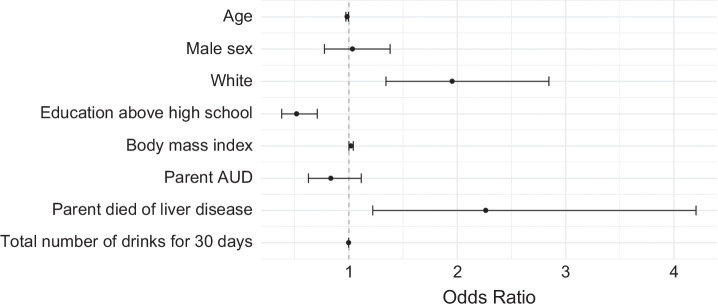
Factors associated with the development of alcohol-associated hepatitis: Estimated odds ratios and 95% CIs from logistic regression analysis. Hispanic ethnicity was also associated with an increased risk of developing alcohol-associated hepatitis (OR=3.14, 95% CI: [1.20, 8.20]; *p*=0.02). It was not included in the figure due to its wide CI.

The effects of factors associated with AH development estimated from the logistic regression model are shown in (Figure 1). There was no significant association between parental AUD and AH risk in the offspring (OR=0.83; 95% CI: [0.63, 1.11]; *p*=0.22). However, parental mortality due to liver disease was found to be associated with a more than doubled AH risk in the offspring (OR=2.26, 95% CI: [1.22, 4.20]; *p*<0.01). Older age was associated with a reduced risk of AH; for each year increase in age, there was a 1.6% reduction in the odds of developing AH (OR=0.98, 95% CI: [0.97, 0.99]). White participants had nearly doubled AH risk compared to non-Whites (OR=1.95, 95% CI: [1.34, 2.84]; *p*<0.01). Individuals of Hispanic ethnicity had a significantly higher risk of being an AH case compared to non-Hispanics (OR=3.14, 95% CI: [1.20, 8.20]; *p*=0.02). Furthermore, individuals with education beyond high school had less than half the risk of developing AH compared to those with high school or lower levels of education (OR=0.52, 95% CI: [0.38, 0.71]; *p*<0.01). Interestingly, patients with AH consumed, on average, fewer drinks in the 30 days prior to study enrollment than HDC (OR=0.998, 95% CI: [0.997, 0.998]; *p*<0.01). BMI was not significantly associated with AH risk (OR=1.02, 95% CI: [0.99, 1.04]; *p*=0.06).

### Factors associated with short-term survival

**FIGURE 2:**
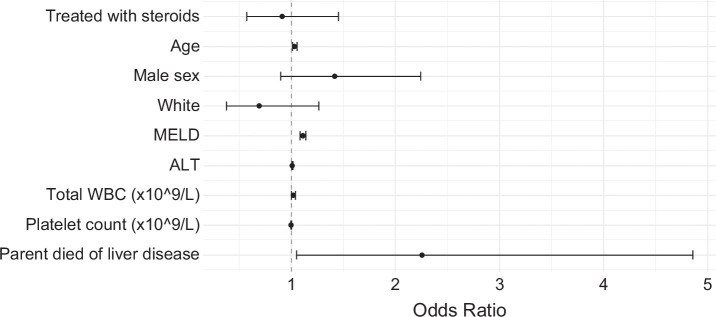
Factors associated with 90-day mortality in patients with alcohol-associated hepatitis in the AlcHepNet cohort: Estimated HRs and 95% CIs from Cox regression.

We further examined the effects of parental AUD and liver disease-related death on the 90-day survival of patients with AH in the AlcHepNet cohort (n=655). The estimated HRs and 95% CIs are presented in (Figure 2). Briefly, parental death due to liver disease was significantly associated with death in the offspring (HR=2.26, 95% CI: [1.05, 4.86]; *p*=0.04), after controlling for the effects of other known risk factors for AH mortality. Other significant factors included age (HR=1.03, 95% CI: [1.01, 1.05]; *p*<0.01), MELD score (HR=1.11, 95% CI: [1.08, 1.14], *p*<0.01), ALT (HR=1.01, 95% CI: [1.00, 1.01]; *p*<0.01), total WBC (×10^9^/L) (HR=1.02, 95% CI: [1.000, 1.04]; *p*=0.05), and platelet count (10^9^/L) (HR=0.998, 95% CI: [0.995, 1.001], *p*=0.22). These results indicate that the existence of parental advanced liver disease may influence AH severity.

## DISCUSSION

While AUD is an essential component of AH, the development and outcome of AH are likely to be the results of multiple clinical, behavioral, environmental, and genetic factors and their interactive effects. A previous study found that patients with alcohol-associated cirrhosis are more likely to report liver disease in a father with alcohol problems.^15^ However, the role of familial factors, either through genetics or upbringing, in facilitating the transition from AUD to ALD has not been carefully studied. In this study, we showed that the risk of developing AH was more than doubled in those with a parent who died of liver disease after controlling for the effects of age, sex, race/ethnicity, education (as a surrogate to socioeconomic status), and current drinking behaviors. This previously unrecognized association points to cross-generational genetic influences that affect both the development and the outcome of AH.

Earlier studies have examined genetic variations associated with AUD and certain types of ALD, such as alcohol-associated liver cirrhosis.^1^,^14^,^26^,^27^,^28^,^29^,^30^,^31^ Our data suggest that parental liver disease mortality significantly heightened the risk of AH in the offspring. Interestingly, the added risk does not appear to be derived, at least not solely, from the same genetic variants that underlie AUD because neither the participant’s own recent drinking behavior nor their parent’s AUD was associated with increased AH risk. Admittedly, physical illness may have forced patients with AH to moderate alcohol consumption. Nonetheless, the data opened up the possibility that either liver disease-related genetic variants have influenced an individual’s susceptibility to AH or the stress and environmental change that come with parental death have led to hazardous drinking in the offspring, which in turn increased AH risk.

Multiple studies have examined the genetic variations in metabolic dysfunction–associated steatotic liver disease and their progression to liver cirrhosis.^32^,^33^,^34^,^35^,^36^,^37^ Both genome-wide association study and candidate gene approaches have been employed in such investigations, the former in identifying genetic risk factors for alcohol-associated liver cirrhosis,^14^ and the latter in assessing AH risk and severity.^16^ These studies, together with those focusing on the genetic correlates to AUD, provide an excellent foundation for future genetic and genomic investigation of AH. However, one should not dismiss the possibility that the ALD risk alleles function differently from the AUD genetic variants, as the latter primarily function through the regulation of alcohol metabolism.

While AH is a condition resulting from excessive and prolonged alcohol exposure, it is the liver’s inability to process consumed alcohol that ultimately leads to tissue inflammation and cell injury. In the present study, parental death due to liver disease was a significant life event that may indicate not only hereditary predisposition but also environmental change. Together, the 2 could create a milieu of complex and interacting influences contributing to the risk of AH development. However, the current study had limited contextual data to discern the relative contributions of the genetic and environmental influences. This question remains to be addressed by future studies.

The lack of association between parental AUD and the risk of developing AH in the offspring, on the other hand, is not entirely surprising for 2 reasons: (1) Epigenetic changes in the parental genome due to chronic alcohol exposure can alter the risk of AH in the offspring without changing the gene sequence.^38^,^39^ Such effects are often complex and may not be adequately captured by simple associations from a cross-sectional analysis. (2) Our use of HDCs as a comparison group may have also contributed to the difficulty in differentiating the effects of parental AUD because both groups have heightened levels of alcohol consumption.

The reported observations, if validated, could have implications for the clinical management of AH. Parental mortality due to liver disease is a measurable trait. Recognizing it as a significant risk factor allows providers to gauge individual AH risk more comprehensively. Patients with the trait should be considered at higher risk for developing AH. Therefore, assessing family history could help identify individuals at increased risk. Targeted interventions, including those through public health programs, should be explored to prevent hazardous drinking before it becomes a reality. For patients with diagnosed AH, care providers can use family history of liver disease mortality to engage patients, raise awareness of the risk of excessive alcohol consumption, and motivate patients to abstain from alcohol.

The current study combined data from 2 prospective multicenter observational studies of well-characterized patients with systematic and standardized family history assessments. The similarity of the target population, experimental design, case definition, assessment instruments, and implementational protocols have allowed us to analyze the combined data with improved analytical power than could be ascertained from the individual studies.

In addition to the hereditary influences, we also observed that (1) older age and a higher level of education were negatively associated with the risk of AH development, and (2) higher MELD scores were associated with increased 90-day mortality in AH cases. These findings were not surprising and consistent with findings from previous studies.

A limitation of the study is the lack of granular details on parental drinking, liver disease, and specific causes of death. Neither TREAT nor AlcHepNet studies have documented the duration, quantity, and frequency of parental drinking or the precise diagnoses of liver disease that had led to death, thus making it difficult to discern the most harmful drinking patterns (eg, drinking during pregnancy) and exact liver diseases contributing to death. Since the patients with AH in the AlcHepNet and TREAT cohorts were relatively young, it was possible that their parents had advanced liver disease but were still alive. The full extent of parental liver disease and mortality on AH development in the offspring remains to be examined in future studies. Finally, we acknowledge the possible influences of recall bias in family history assessment, especially in AH cases, whose illness may prompt them to a more rigorous recall of their parent’s illness and death.

In summary, our data support hereditary and possibly environmental influences on the development of AH. Assessing the family history of liver disease-related death may help identify people at higher risk and facilitate discussions on mitigating the risk by reducing alcohol consumption. Further studies are needed to evaluate the genetic, environmental, and epigenetic factors underlying AH development.

## Supplementary Material
