# Supplementary material for: Parental liver disease mortality is associated with unfavorable outcomes in patients with alcohol-associated hepatitis
Source: Hepatol Commun. 2025 May 23;9(6):e0666. doi: 10.1097/HC9.0000000000000666 (PMC12106198; doi:10.1097/HC9.0000000000000666)
Supplement: Supplementary file 1 [file hc9-9-e0666-s001.docx]

Supplementary Table 1. Demographic and clinical characteristics of the TREAT and AlcHepNet Cohorts

| variable label | Overall N=1356 | TREAT N=459 | AHN N=897 |
| --- | --- | --- | --- |
|  |  |  |  |
| Age at enrollment | 45.9 ± 11.5 | 45.9 ± 11.4 | 45.9 ± 11.5 |
| GENDER |  |  |  |
| - Male | 801 (59.6%) | 285 (62.1%) | 516 (58.3%) |
| RACE |  |  |  |
| - Nonwhite | 250 (18.4%) | 70 (15.3%) | 180 (20.1%) |
| - White | 1106 (81.6%) | 389 (84.7%) | 717 (79.9%) |
| ETHNICITY |  |  |  |
| - Hispanic or Latino | 70 (5.3%) | 10 (2.2%) | 60 (6.9%) |
| - Non-Hispanic | 1250 (94.7%) | 442 (97.8%) | 808 (93.1%) |
| Highest Level of Formal Education Completed |  |  |  |
| - Elementary/year10/High school | 492 (38.5%) | 172 (38.4%) | 320 (38.6%) |
| - Trade School/College/Graduate program | 786 (61.5%) | 276 (61.6%) | 510 (61.4%) |
| Are you currently employed? |  |  |  |
| - No | 542 (61.2%) | 28 (60.9%) | 514 (61.2%) |
| - Yes | 344 (38.8%) | 18 (39.1%) | 326 (38.8%) |
| BMI | 29.2 ± 7.3 | 29.1 ± 7.2 | 29.3 ± 7.4 |
| Has your blood or natural father/mother been an alcoholic or problem drinker at ANY time in his/her |  |  |  |
| - No | 515 (41.8%) | 179 (41.2%) | 336 (42.1%) |
| - Yes | 718 (58.2%) | 255 (58.8%) | 463 (57.9%) |
| Did he/she die of liver disease? |  |  |  |
| - No | 1076 (93.1%) | 384 (90.6%) | 692 (94.5%) |
| - Yes | 80 (6.9%) | 40 (9.4%) | 40 (5.5%) |
| Indicate the total number of drinks for 30 days | 225.1 ± 243.6 | 310.3 ± 275.6 | 176.8 ± 208.6 |
| Indicate the total number of drinking days out of the 30 days prior to the visit | 19.9 ± 10.7 | 24.7 ± 7.8 | 17.2 ± 11.1 |

Supplementary Figure S1. Derivation of the study cohort
